# Critical interloping, collaboration, and entangling the ‘expert cultures’ of art, science and lived experience: Jacqueline Donachie’s artist-led research on myotonic dystrophy

**DOI:** 10.1136/medhum-2026-013979

**Published:** 2026-08-14

**Authors:** Fiona Johnstone

**Affiliations:** Durham University

## Abstract

The article positions artist-led research as a vital methodology for an engaged, transformative and translational medical humanities. Recent scholarship aligned with the critical visual medical humanities has drawn attention to art practices as epistemologically generative, suggesting that the methods of criticality mobilized by artists offer a distinctive and valuable approach to addressing questions of health. In line with this approach, this article proposes artist Jacqueline Donachie’s sustained collaborative enquiry into the science and lived experience of myotonic dystrophy as a compelling example of how artist-led research can reshape the way scientists and clinicians engage with and understand a particular condition. Donachie’s practice demonstrates how artistic methods – dialogue, observation, ethnographic engagement, and aesthetic distillation – can productively entangle scientific and experiential knowledge, foregrounding the perspectives of families and patients whose insights have historically been marginal to genetic research and clinical care. By bringing different expert cultures together in mutually generative conversation, Donachie’s practice raises important questions about how different forms of knowledge are mobilised, and how they might inform and enrich one another. The article contributes to ongoing efforts to conceptualise collaborative knowledge-producing practices across the (visual) medical humanities by advancing the figure of the critical interloper, a practitioner who works between epistemic spaces, drawing together diverse knowledge systems in ways that challenge, unsettle and ultimately bring about change.

‘We didn’t listen to the families.’ One of the most poignant lines in the film *Tomorrow Belongs to Me* (2006), produced by the artist Jacqueline Donachie in collaboration with geneticist Darren G. Monckton, is spoken by Stephanie L. Sherman, one of the first genetics researchers to theorise a biological mechanism for the anticipatory inheritance pattern of fragile X syndrome, a genetic neurodevelopmental disorder which occurs with increasing frequency in each generation of a family.[1] Prior to Sherman’s hypothesis, anticipation (which characterises a number of genetic disorders including fragile X, myotonic dystrophy and Huntingdon’s Disease) had been regarded as statistical rather than biological, explained as a consequence of bias in the way that data from affected families was reported and studied. Donachie and Monckton’s 18-minute film focusses on a series of paradigm-shifting breakthroughs in genetics research: the driving narrative is the thrill of discovery and the advancement of scientific knowledge. At key moments, however, the perspectives of families living with anticipatory genetic conditions disrupt and complicate what might otherwise register as a straightforward celebration of medical progress.

*Tomorrow Belongs to Me* is one of several outputs (a film, publication and exhibition) developed during an extended collaboration between Donachie and Monckton. When three generations of Donachie’s own family were diagnosed with myotonic dystrophy – a type of muscular dystrophy, a genetic disorder which causes progressive muscle loss and weakness – Donachie mobilised her skills as an artist to broker conversations with genetics experts in order to find out more about the condition. These initiatory events are explored in the artist’s book *DM* (2002).[2] The ensuing project with Monckton brought together distinct but complimentary forms of expertise, interweaving the lived experience of Donachie’s family members with insights from pioneering genetics researchers. A decade later, Donachie worked with women recruited through the UK Myotonic Dystrophy Patient Registry to explore relationships between sisters where one is living with myotonic dystrophy and the other is unaffected: the resultant film *Hazel* (2016) extends the themes of family resemblance, genetic inheritance, illness, disability and care introduced in the earlier works.[3] Together these works comprise a significant body of artist-led research on myotonic dystrophy, shaped by ongoing dialogue with different expert communities over many years.

This article proposes Donachie’s work as a compelling example of what practice-led[4–6] or artist-led research can accomplish in the field of medical and health humanities.^i^ It argues that by bringing different ‘expert cultures’^ii^ together in mutually generative dialogue, Donachie’s practice raises important questions about how different forms of knowledge – clinical and experiential, professional and personal, artistic and scientific – are mobilised, and how they might inform and enrich one another. Early work in the medical humanities (operating in a ‘supportive friend’ capacity to clinical training and practice) was sometimes reluctant to recognise art as a valid form of knowledge in its own right, often side-lining artists and their practices into illustrative, pedagogic or art-therapeutic roles.[7,8] More recently, however, scholarship aligned with the critical medical humanities has drawn attention to art practices as epistemologically generative: see for example, a newly published volume of essays on *Art and the Critical Medical Humanities*, which explores ‘how the methods of criticality mobilized by art practices which operate in art worlds offer a distinctive and valuable approach to addressing vital questions of human health and flourishing’ (Johnstone, Morehead and Wiltshire 2026, p.7).[9] In line with this approach, this article positions Donachie’s work on myotonic dystrophy as a case study in how artist-led enquiry can reshape the ways health researchers and health workers engage with and understand a particular condition.

## Critical interloping

This article contributes to ongoing efforts to conceptualise experimental and collaborative knowledge-producing practices across the medical humanities by advancing the figure of the *critical interloper*: a practitioner who works between epistemic spaces, drawing together diverse knowledge systems in ways that challenge, unsettle, and ultimately bring about change. The term ‘critical interloper’ is borrowed from Stella Bolaki’s book on art and medicine, *Illness as Many Narratives*, where Bolaki describes her own interdisciplinary project as participating in a twofold ‘critical interloping’:

inserting a variety of artistic and cultural representations that explore illness within the field of the medical humanities to expand its scope and existing approaches, and to create a more inclusive illness narrative canon; and at the same time modelling ways in which the arts and arts/media scholarship can enlarge their practices and critical approaches […] through more explicit dialogue with the critical medical humanities. (Bolaki 2016, p.13)[10]

For Bolaki, this kind of interloping is ‘bridge-building’: it is *critical* because it does not simply add to an existing range of texts or artworks, but brings together different methodologies and epistemologies to actively question and transform current practices and paradigms. Critical medical humanities frames itself as explicitly interdisciplinary and ‘entangled’ as well as ‘engaged’ with different communities: dialogue across different epistemic groups and the aspiration towards transformation are vital for meaningful work.[11–15] Using Donachie’s work as a case study, this article argues that the processes and practices of artist-led research can be particularly well-suited to the two-fold task of building bridges between epistemic communities and working towards genuinely impactful outcomes.

Bolaki’s theorization of critical interloping can be usefully developed by considering two further conceptual mobilizations of the interloper figure. In his book *The Interloper: Lessons from Resistance in the Field*, sociologist Michael Anteby uses the interloper to explore obstacles typically encountered in field research (including challenges in negotiating access to the field) and in doing so to reframe them as intellectually productive (in revealing something about the constitution of that field). Anteby suggests that fieldworkers are exemplary interloper figures: ‘individuals who intrude in places, situations or activities and disrupt the status quo by examining other people’s affairs, even when trying to blend in’ (Anteby 2024, p. 4).[16] Although Anteby is not specifically writing about artists, the questions that he raises – particularly around access to the field and the insider/outsider status of the embedded researcher – have also been discussed in the literature that addresses the affinities between artistic and anthropological/ethnographic practice.[17–21] Field-research is a key aspect of Donachie’s practice (and of many other artists conducting health research: further UK-based examples might include Catherine Baker, Beverley Hood, Sofie Layton, Liz Orton, Deborah Padfield, and Ilona Sagar). By recognising artist-interlopers as fieldworkers (in Anteby’s sense), we can understand critical interloping as an interdisciplinary practice that goes beyond mutually enriching dialogue to actively intervene, disrupt, or bring about change.

The figure of the interloper also facilitates a consideration of how disciplinary boundaries are maintained, challenged and eventually transformed. Journalism scholar Scott Eldridge uses the concept of ‘interloper media’ to describe new digital media actors (such as WikiLeaks) ‘who claim to belong to the journalistic field while also openly criticizing traditional media and journalists they associate with institutions of power.’ (Eldridge 2019, p.8).[22] The concept of interloping thus raises questions about who is considered to be ‘inside’ or ‘outside’ a given field, discipline or epistemic community, and the processes through which those groups construct their own norms and standards. Such questions are a foundational concern of Science and Technology Studies (STS), which analyses how heterogenous actors with different standards of evidence, values, and practices work together to produce knowledge: laboratory and field studies show that collaboration often requires translation and negotiation between distinct ‘epistemic cultures’,[23] while actor-network theory demonstrates how shared facts emerge through networks that connect scientists, instruments, institutions, and non-scientific actors.[24,25] The concept of the critical interloper shares some commonalities with the STS concepts of boundary objects and boundary work[26,27] as well as Galison’s concept of trading zones.[28] However, there are also some important differences. Broadly speaking, boundary objects account for the stabilisation of collaboration across epistemic difference, while trading zones explain how coordination can occur without shared language or consensus. The figure of the critical interloper occupies the space between and across these models, foregrounding an agentive mode of engagement that does not merely mediate or traverse disciplinary boundaries, but actively interrogates and seeks to transform the epistemic, institutional, and political conditions under which interdisciplinary collaboration takes place.

Scholars including Hannah Star Rogers, Megan Halpern and Silvia Casini have recently argued for the value of introducing art into STS.[29–32] The field of ASTS (Art, Science and Technology Studies) recognises that ‘knowledge produced through artistic pursuit has been subjugated, devalued and undermined’ (Rogers and Halpern 2021, pp.9-10).[29] ASTS counters this by treating art practice as research and calling for artists and scientists to resist instrumentalization of the arts in the service of science, emphasising shared commonalities in creative and epistemic practices, including ‘image making’, ‘object making’ and the material-rhetorical concept of the ‘experiment’ (Rodgers 2023, p.64).[30] Rogers and Halpern suggest that ‘more work is needed to fully conceptualize questions around collaborations’ (2021, p.39)[29]. This article furthers that aim by reading Donachie’s work not only as a *product* of collaboration, but as a critical reflection on the *processes* of collaborative research.

In the section that follows, I briefly outline some of the tensions of art-science collaboration that have been articulated to-date, locating Donachie’s work in the historical context of the funding environment of the late 1990s and early 2000s. I then reflexively situate my own approach to this material as an art historian working across contemporary art practice and (visual) medical humanities.^iii^

## On collaboration: challenging the sci-art paradigm

Donachie’s work on myotonic dystrophy aligned with an increasingly supportive funding environment for art–science collaborations, and a period marked by heightened public interest in genetics. The Human Genome Project launched in 1990, and was declared complete in 2003, with 92% of the genome mapped and sequenced. Unsurprisingly, a substantial body of artistic practice from this moment engages directly with developments in science[33–35], and particularly genetic science.[36] The influential SciArt scheme run by the UK-based biomedical research funder the Wellcome Trust from 1996-2006 (initially as part of a consortium of funders)^iv^ aimed to support ‘visual arts projects which involved an artist and a scientist in collaboration to research, develop and produce work which explored contemporary biological and medical science’. The scheme has been widely credited with consolidating and amplifying a set of practices already taking shape by the mid-1990s, when artists were increasingly seeking out collaborations with scientists.[37] Over its ten-year duration, the programme supported 118 projects with nearly £3million of funding: Donachie’s work with Monckton was directly supported through a Research and Development (R&D) Award (which made the initial collaboration possible) and a subsequent (more generous) Production Award for *Tomorrow Belongs to Me*. A photograph of the installation of *Tomorrow Belongs to Me* at the Hunterian Museum in Glasgow in 2006 is reproduced in the opening pages of the Wellcome Trust’s official SciArt evaluation, suggesting that the project may have been regarded by the Trust as a particularly successful example of art-science collaboration supported by their funding strategy.

However, critics of SciArt (the funding scheme) and ‘sci-art’ (a label for a broader category of work) have suggested that in practice its supposed interdisciplinarity often slipped into science communication, sacrificing the critical potentiality of art, fetishizing technology, and ignoring the structural biases and asymmetric power relations between scientists and artists.[38] The Wellcome Trust’s evaluation of the SciArt programme, whilst broadly celebratory in tone, recognized the branding issues associated with the ‘SciArt’ label, changing the scheme’s name to ‘Arts Awards’ when it was relaunched in 2007. The report noted that several interviewees voiced concerns about the associations that had quickly become attached to the term *Sciart*. What began as the name of a specific funding scheme was seen to have evolved into a broad — and often inaccurately used — label for a supposed genre of artistic practice. Many felt this shift had negative consequences, notably that the term contributed to an unhelpful and sometimes unfair marginalisation of art engaging with scientific themes (Glinkowski and Bamford 2009, p.29).[37] Donachie has voiced similar concerns about the ‘sciart’ label, noting ‘discussions around the interface of two worlds have been questioned, reported and theorised, but quality of artwork has not always been at the forefront of this’ (Donachie 2016, p12).[39] The end result of an artist-led enquiry should not be merely the communication of complex scientific information. This is succinctly expressed by Donachie in a statement in a catalogue text: ‘Working with research the end result is not information […] the end result is art’ (Donachie in Jeffrey 2016, p.50).[40]

Given this article’s interest in the co-constitution of different types of expertise, it feels appropriate to briefly outline my own positioning and methods. I am trained as an art historian (although increasingly feel constrained by the narrowness of that as a disciplinary label, and on a day-to-day basis work mainly alongside colleagues from other fields). I began exploring Donachie’s work as part of a larger project on artists who carry out collaborative research in healthcare or biomedical settings: I interviewed Donachie, examined the copy of her artist’s book *DM* (2002) held by the Wellcome Collection in London, and watched her two films, *Tomorrow Belongs to Me* (2006) and *Hazel* (2016) on my laptop. I also read the secondary literature on her work (mainly exhibition catalogues) as well as Donachie’s practice-based PhD thesis (2016). My approach recognises art as an expert culture and a form of knowledge-making in its own right. While this article situates Donachie’s work as artist-led medical humanities research, it does not limit its analysis to explaining how the art might be ‘useful’ to clinical practice (for example). Rather, I am interested in how collaborative work happens between artists, patients and their families, and clinicians and biomedical researchers, and particularly in the friction points where the norms and expectations of ‘art worlds’ do not neatly align with those of biomedical research or clinical practice.

As an art historian, I have been trained to focus my analysis primarily on art objects; that is, on the end-products of what is often an extensive process of artistic research. In writing this article, I have become aware of the limitations of this approach, which misses what is often most interesting and valuable about artists’ practices in the contexts of medical and health humanities. Whilst remaining attentive to the artistic outputs, therefore, this article also considers the *processes* of artist-led research as epistemologically generative. As Flora Lysen notes in their own study of art-science collaboration, the ‘A’ in ASTS signifies art not only as an object of analysis but also as a form of methodological innovation within science and technology studies (Lysen, 2023, p.220).[41] Building on the work of scholars such as Lysen, Rodgers and Casini in bringing art into STS, this article proposes a parallel intervention in medical humanities, positioning art as both an object of study and a methodological resource.

The remainder of this article offers a close reading of successive phases in Donachie’s research on myotonic dystrophy. It begins with an analysis of the artist’s book *DM* (2002), which documents her family’s diagnostic experience and traces the origins of her collaboration with Darren Monckton. This is followed by a discussion of *Tomorrow Belongs to Me*, an extended collaborative project (2001–2026) that generated a film, publication, and exhibition. The final section examines *Hazel* (2016), marking a shift away from genetic science as a primary focus towards the embodied and social experiences of middle-aged women living with myotonic dystrophy. The article concludes by reflecting on the methodological limitations of the single-artist case study and outlining the need for a more expansive and comparative programme of research in this field.

## *DM* 

**Figure 1:**
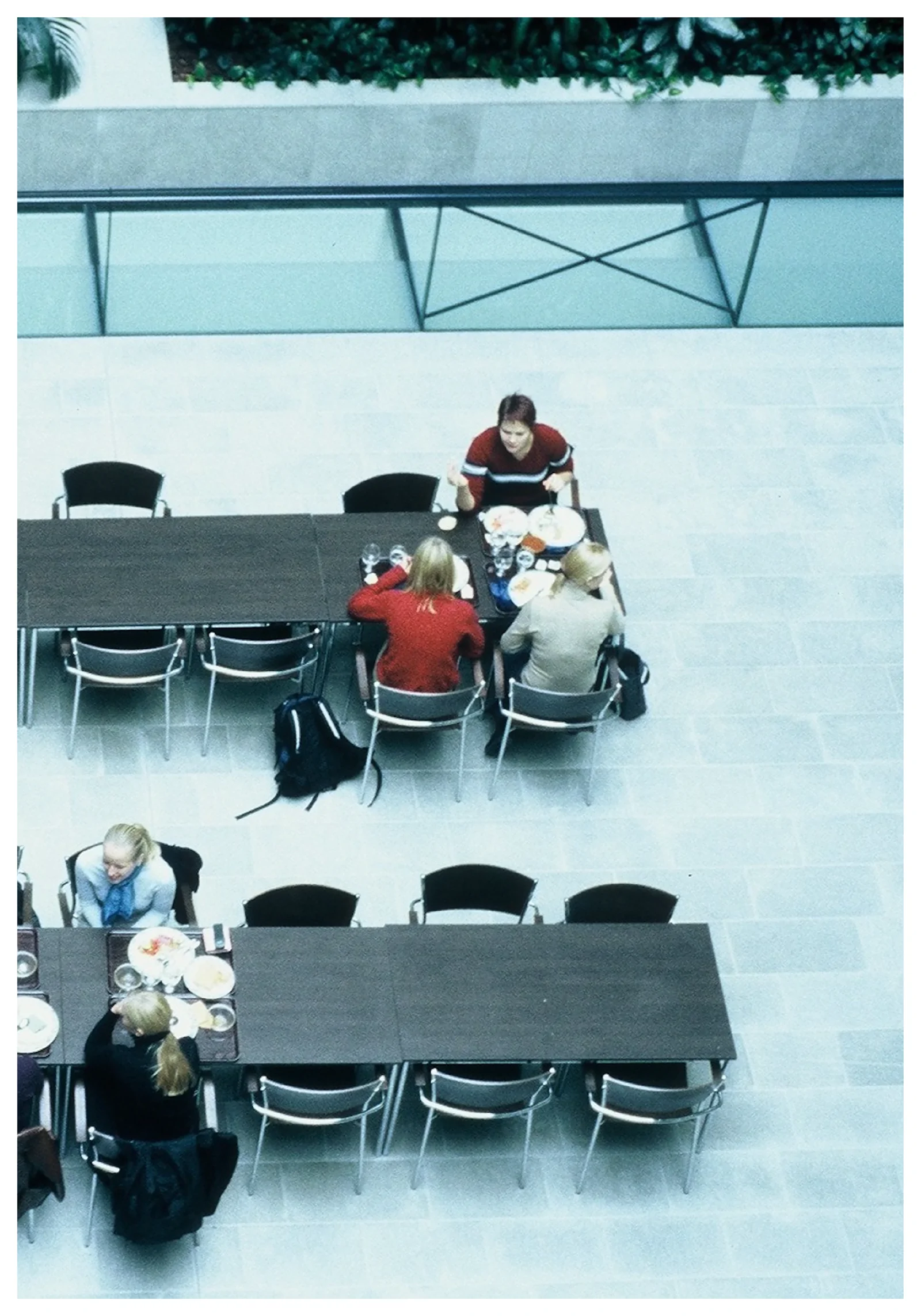
Jacqueline Donachie, *DM* (2002). Artist’s book. Image © the artist.

The beginnings of Donachie’s collaboration with Monckton and other biomedical researchers is recounted in the artist’s book *DM* (2002) [Figure 1], which is simultaneously a story of a family’s collective diagnosis, documentation of a research process, and an invitation to reflect on how different forms of research – artistic and scientific, personal and professional – co-exist and mutually inform each other. A relatively modest-sized, A6 cardboard-bound, multiple-edition volume of approximately 40 pages, its contents are divided into three sections, comprising a first-person text by the artist, 31 captioned photographic portraits of genetics researchers and members of the artist’s family, and an enigmatic final section of three photographs of disconcertingly uninhabited hospital-like rooms and a fourth photograph of a hand holding a pen to a blank sheet of paper.

Donachie describes the first-person text of the opening section as ‘not narrative […] more like poetry […] it was not originally written as text for a book, but for myself, to clarify what was going on for us at a difficult time’ (Donachie 2016, p13).[39] The opening line establishes the close relationship between Donachie and her sister and the motif of family resemblance that threads through this work:

All my life I’ve been told how much I look like my sister.[2]

People used to take them for twins, although they never saw the similarity themselves. Both sisters were pregnant at the same time, Donachie with her first, her sister with her second. Her sister’s baby was born first, almost 8 weeks prematurely and extremely unwell. Following ‘samples and tests and lots of questions’ it transpires that the baby had inherited ‘something’ from her mother (as well as her hazel eyes); later on, they find out that her sister had inherited ‘this thing’ from their father. Because ‘this thing’ is genetic, Donachie and her unborn baby are identified as at risk. A page later, after ‘more tests and more waiting and more kicks from an ever-fattening belly’, Donachie is told that she does not have ‘the thing that they had’ and that she cannot pass it on to her own child.

In the months that follow, multiple members of the family across all three generations are diagnosed with ‘this thing’. The refusal to name the condition has a variety of effects, contributing to the lyrical qualities of the text (which in its rhythm and tempo shares stylistic similarities with a children’s storybook or fairy-tale) and rendering the condition a shadowy presence which is powerfully felt but as yet invisible and unknowable. Rather than leading with the clinical details of the diagnosis, the text initially focusses on the affective textures of the shared diagnostic experience. Suspense and shock are invoked by a print layout which sets a single line of unresolved text on an otherwise blank page. The isolated phrase ‘Then my sister had her baby’ feels dark and ominous; the verso of the page resolves the narrative tension with deadpan understatement: ‘The baby wasn’t so well.’ Other shared responses include stress (‘round this time my mum started to smoke again’), and even dark humour (‘we all started to notice funny wee things like the fact that Susan could no longer walk on her tiptoes, which made for a laugh as we all tried it, up and down the kitchen floor at my parent’s house’).

Critical diagnosis scholar Annemarie Jutel observes that ‘it is impossible to separate diagnoses from the stories they impart’ (Jutel 2023, p.300).[42] A diagnosis is frequently understood as a defining moment in a person’s life: when the condition identified is serious, it can mark a profound transition, dividing life into a ‘before’ and ‘after’. In *DM*, the family’s diagnosis is marked by a shift in the narrative voice from the individual first-person pronoun ‘I’ to the collective ‘we’: ‘Since that year we’ve found out more about this thing that they all have’. *DM* is an unusual illness narrative in telling the story of a family rather than a singular individual.

Stuart Murray notes how, within a Euro-American tradition of health narratives, the prevailing modes of storytelling are ‘predominantly about personal experience, reflecting the stress such cultures place on the role of the individual and apparently centred self’ (Murray 2023, pp.13-14)[43]. Murray further suggests that while such stories ‘usually revolve around how one figure encountered illness or disablement […] this is not how health and disability are always understood: in fact, they are relational in so many ways’ (Murray 2023, p.14).[43] *DM* foregrounds the relationality of illness and disability: this begins with shared family experience (foregrounded in the first-person text that comprises the opening section of the book), extending in subsequent sections to invoke the perspectives of researchers, clinicians, counsellors, technicians, charity workers and volunteers, and other affected families.

The story shifts to a practical account of the unusual genetic inheritance pattern of ‘the thing’ and the chromosomal mutation that causes it:

It grows and repeats itself through your lifetime, and the severity of the symptoms multiply as it passes through the generations. The type that my niece got from my sister is worse than the type my sister got from my dad. […]

The repeating is all at a very tiny level, in one particular chromosome, so it’s not really visible to anyone other than a lab technician with a sample of your DNA. […][2]

The complexity of the condition necessitates contact with increasing numbers of medical professionals, foregrounding the co-constructed qualities of illness experience: ‘You meet a lot of people’.

Although *DM* is a record of a collaborative research process, the clinical collaborators remain unidentified in the first-person text of the opening section. Initial connections with scientists are recounted in the most impersonal of terms: ‘I began to work with some people who did a lot of research into the gene itself, and they gave me lots of scientific papers to read’. The effect is a flattening of hierarchies, an investment in shared exploration rather than a reification of particular forms of expertise. Reading these scientific papers, the artist learns about a place in Quebec, Canada that has an instance of disease ten times that of the national average: this is known as ‘a strong founder effect’. There was a theory that everyone who has the disease are descended from the same family. Donachie wonders:

So if we went there, my sister and I, would we look familiar?[2]

The family’s diagnostic narrative transforms into an extraordinary research trip, ‘seven days, six flights. One professor, one doctor and two sisters’, and the account turns to the challenges and rewards of interdisciplinary art-science collaboration:

The geneticists marched us all off to the Science Museum and later that day I dragged them to the Museum of Contemporary Art. We were sceptical but respectful of how each of our worlds were portrayed, but felt better for this unplanned bonding.[2]

The sisters, the doctor and the geneticist meet a lot of people, ‘gave out a lot of highland toffee’ and ‘drew family trees on dinner placemats’. They meet families who are worst affected (which ‘scared the shit out of my sister and I’) and families less affected (which ‘made us feel good’). They eat blueberries with two sociologists who study the cultural effects of the condition on affected families, and swim in a lake with an eminent neurologist and his genetic counsellor wife. They do not find the ‘recognition’ or ‘likeness’ they are searching for.

The concepts of ‘likeness’ and ‘recognition’ are taken up in the second part of the book which consists of photographic portraits: staff at the Muscular Dystrophy Association in Canada; a PhD researcher in Molecular Genetics at the University of Glasgow; three young blond women sitting at computers in a sterile white office space are identified as ‘Researchers, Biomedical Institute, Helsinki University’. The first image clearly taken in a domestic (as opposed to professional) environment is of the artist’s mother, a smiling woman with short grey hair, dressed in a white sweater with glasses hanging at her neck. In the foreground are a bunch of red tulips; in the background, a white wooden door, a hallway and staircase. The caption identifies her by name, profession, and family members: ‘Kathleen Donachie, Employment Liaison Officer, Glasgow. Husband, two children and two grandchildren affected’. On subsequent pages, between portraits of consultants in medical genetics, we meet the artist’s sister, ‘Susan Craig (with nephew Archie), Software Technician, Department of Computing and Electrical Engineering, Herriot Watt University, Edinburgh. Diagnosed May 1999’; the artist’s father, standing next to a patch of daffodils; and a tiny bonneted baby in a neonatal intensive care unit, ‘Rhona Craig, born congenitally affected, May 1999’. The captioning and consistent style and size of image afford affected individuals and their family members as much status as professional researchers, and many of the portraits have a snapshot-like quality: the effect is an extended family album of individuals joined by ‘this thing’.

Part three of the book consists of four images. The first three are of empty spaces: a row of chairs lined-up next to a lift door suggest a clinical waiting room. The final image is a closely-cropped photograph of a hand holding a pen to a sheet of A4 paper, with the camera (and therefore, the viewer) positioned across a small table from the person holding the pen. A discarded knife, teaspoon and used catering pod of milk lie in the foreground: one person is clearly trying to explain something to another. The sheet of paper has marks on it, but the meaning is unavailable to the viewer. This final image sits opposite an informational text (‘Myotonic dystrophy (DM) is the commonest form of muscular dystrophy in adults […] there is currently no cure’) and the contact address for three charities, the Muscular Dystrophy Campaign, the Myotonic Dystrophy Support Group, and the Scottish Muscle Network. Like the blank sheet of paper in the final image, *DM* resists narrative resolution, suggesting instead research questions yet to be posed and challenges still to be (collectively) worked out and worked towards. My experience of viewing the copy of *DM* held by the Wellcome Library in London was both perplexing and rewarding; it felt like an intimate act of witnessing, but also demanded that I proactively create my own connections between texts and images, protagonists, experiences and ideas.

*DM* poeticises and distils the mechanics of interdisciplinary collaboration. Curious about how such collaborations work in practical terms, I asked Donachie about her experience. Following her family’s diagnosis, Donachie approached the Division of Molecular Genetics at the University of Glasgow with an enquiry about working together. Successful collaborations are often fuelled by the serendipity of interpersonal connection: Donachie and Monckton met, clicked, and swiftly co-applied for a Research and Development Award through the Wellcome Trust’s Sci-Art Funding Scheme. Donachie recalled Wellcome’s interview process: ‘we had to go down to London […] they basically decided on the chemistry of the two people, whether it was worth giving them the money to go off and work together, which is absolutely how it should be.’[44] For the interview, Donachie and Monckton decided that Donachie would talk about the science and Monckton would talk about the art, thereby evidencing a genuine interest in and engagement with each other’s work: as Donachie observed, ‘you have to be interested in each other […] it’s not enough if the artist wants to look at what the scientist does and the scientist shows off’.[44] The strategy paid off and the funding was used to support a short residency for Donachie in the Department of Human Genetics at the University of Glasgow, followed by the field trip to Quebec 2021.

This emphasis on interpersonal connection is highlighted by the jacket image, a colour photograph of an institutional dining space identified as the central atrium of the Biomedicum, Helsinki, a research and teaching centre located on a hospital campus. Taken from a high vantage-point – perhaps a balcony or raised walkway – the camera focuses on two long canteen-style tables and uniform grey and metal chairs. Groups of people with trays of food gather at the tables, some gesticulating, others leaning forward to listen more closely; bags and backpacks are slung on the floor and coats draped over the backs of chairs; the impression is one of animated discussion between co-workers. Rather than a perhaps more obvious picture of a biomedical research lab – white coats and petri dishes – the image wrapped around the book emphasizes research as a shared venture underpinned by interpersonal connectivity.

Introducing a special issue on artists’ books for the *Journal of Medical Humanities* in 2020, Stella Bolaki observed that the artist’s book ‘has not been explored systematically within the medical humanities and arts in health fields’ (Bolaki 2020, p.2).[45] Despite valuable and substantial scholarship by Bolaki [10,46,47] and more recently Darian Goldin Stahl [48], the full potential of what artists’ books (and artist-led research more broadly) might bring to the study of health remain underexplored and underutilised. Bolaki argues that artists’ books (and by extension, artists’ practices) have the potential to reshape what we know about health in ways that ‘extend beyond narrative legitimacy or a form of struggle against the medical gaze’ (Bolaki 2016, pp.21-22).[11] Darian Goldin Stahl similarly suggests that artists’ books ‘point towards a more collaborative and creative future in health research’ by facilitating ‘critique, dialogue, and learning to enact meaningful change’ (Stahl 2024, p.4).[48] The research processes documented in *DM* epitomise the transformative epistemic qualities of artists’ practices articulated by Bolaki and Stahl, foregrounding the collaborative pursuit of knowledge and breaking down the perceived binary boundary between patient experience and clinical expertise to invoke a more nuanced and potentially impactful encounter. The next section of this article considers Donachie and Moncton’s subsequent project *Tomorrow Belongs to Me* to address in further detail the affordances of artistic research methods for productively entangling patient experience and clinical expertise.

## Tomorrow Belongs to Me

**Figure 2:**
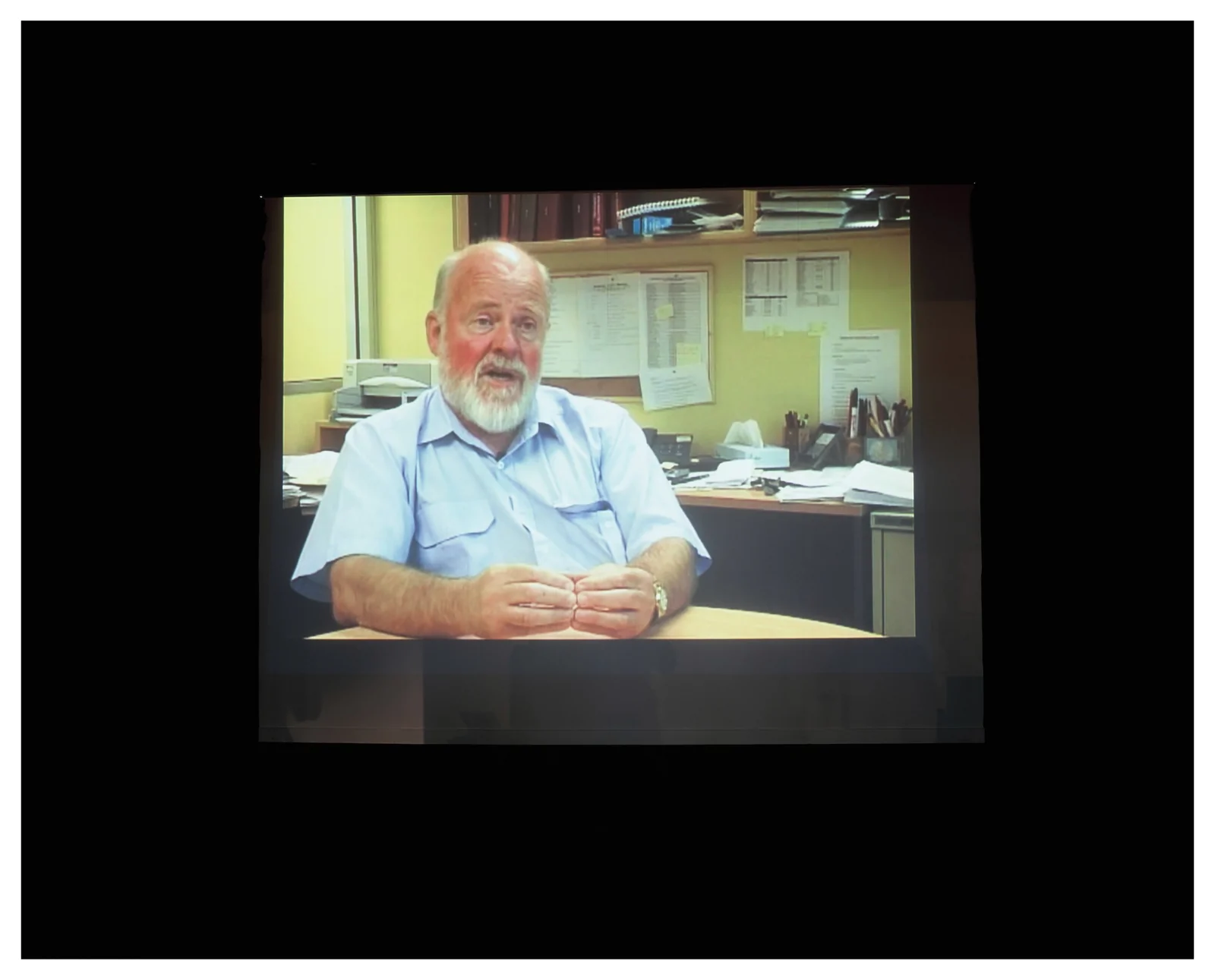
Jacqueline Donachie and Darren G. Monckton, *Tomorrow Belongs to Me*, 18-minute digital video, 2006. Image © the artist. *Tomorrow Belongs to Me* can be viewed on the following link: https://discovery.dundee.ac.uk/en/publications/tomorrow-belongs-to-me/ [accessed 24 April 2026].

**Figure 3:**
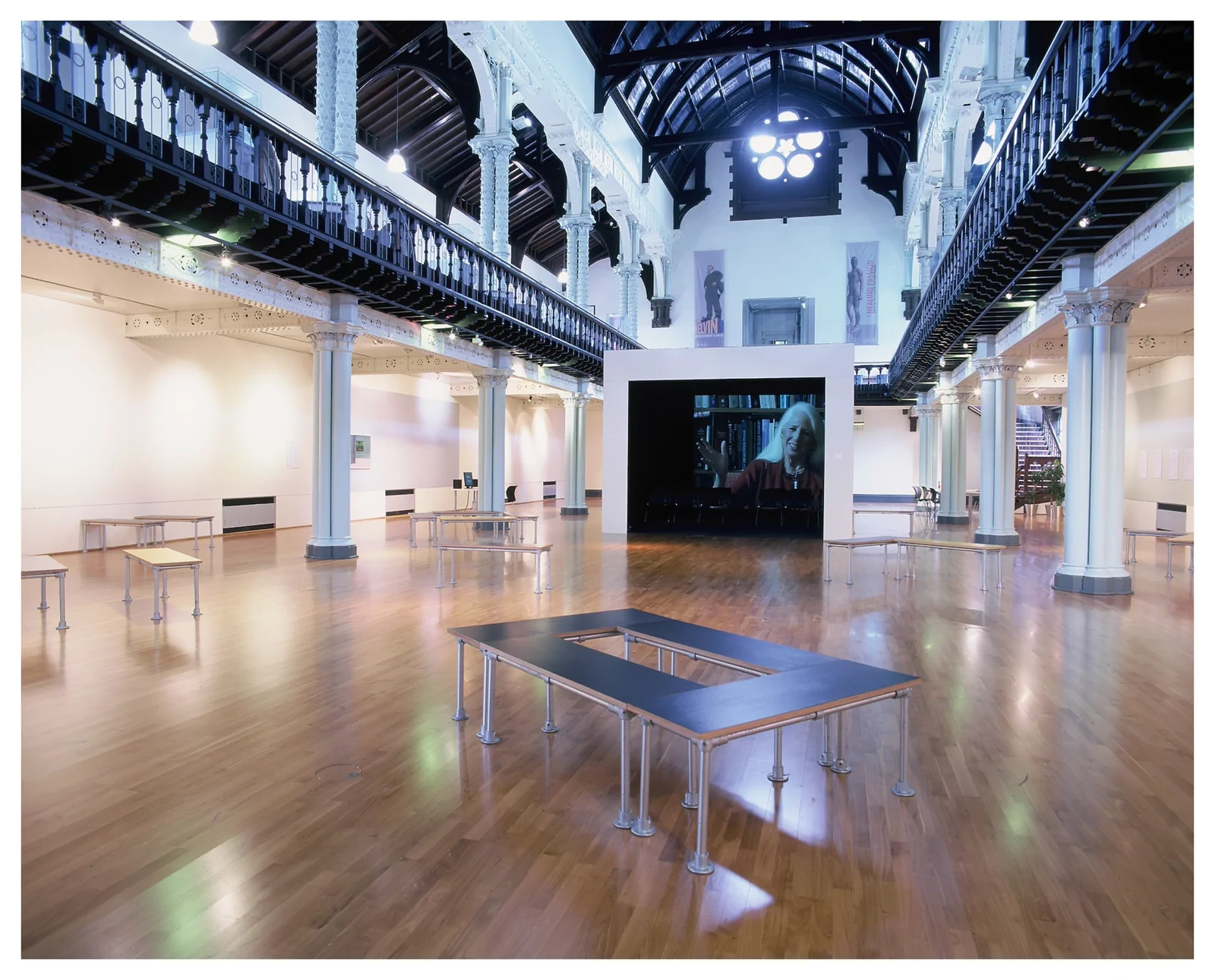
Exhibition installation view, *Tomorrow Belongs to Me New Work by Jacqueline Donachie made in collaboration with Darren G. Monckton*, Hunterian Museum and Art Gallery, Glasgow, 2006. To back of installation: Jacqueline Donachie and Darren G. Monckton, *Tomorrow Belongs to Me*, 18-minute digital video, 2006. In foreground: Jacqueline Donachie, *Tell me about your father’s side*, lacquered wood and steel benches, 2006. Photography Alan Dimmick. Image © the artist.

*Tomorrow Belongs to Me* names an extended collaboration between Donachie and Monckton, building on the experiences and relationships articulated in *DM*, and culminating in a film, exhibition and publication of the same title (all 2006). Like *DM, Tomorrow Belongs to Me* weaves together Donachie’s own family experience with insights from genetics experts. However, the project is much less explicitly autoethnographic than *DM*, and is largely focussed on a story of scientific discovery. The 18-minute film [Figures 2 and 3] is based on to-camera interviews with eleven pioneering genetics researchers speaking from different countries and continents; these interviews were conducted by Donachie and Monckton (and filmed by cameraman Holger Mohaupt) over a fifteen-month period from early 2004 to May 2025. The formidable task of gathering this material is captured in the accompanying print publication via a series of extracts from the artist’s diary which entertainingly reflect on the challenges of balancing an exacting international filming schedule with the demands of a growing young family. Process is similarly made visible on the publication’s inner flyleaf, which is printed with images of Monckton’s boarding passes for international flights. These details make manifest the methods of enquiry that inform Donachie’s art-making process, including communication, observation, and extended dialogue with different expert communities, underscoring the relational and interpersonal nature of the work. The film presents these very qualities as key methods for scientific research.

When transcribed (to help with the editing process) the interviews (totalling around 22 hours of footage) came out at 90,000 words. The accompanying print publication includes 174 pages of edited interview transcripts, representing a significant resource for future research on the history of medicine.[49] Tightly edited from this extensive mass of documentation, the film is a fast-paced and thrilling account of the discovery of the biological mechanism that finally explained the perplexing phenomenon of anticipation. As explained in the film’s opening moments, ‘anticipation’ describes how certain genetic disorders (including myotonic dystrophy, fragile X and Huntington’s disease) become more severe and/or appear earlier or more frequently in successive generations. A typical pattern of anticipation in a three-generation family with myotonic dystrophy might involve a severely affected child with problems from birth; the clinical team would then look at the family and realise that the mother had some milder features of myotonic dystrophy. Going back a generation further, a grandparent might be found to have cataracts but no obvious muscular problems. Until the late 1980s, prevailing scientific opinion held that anticipation was a merely a reporting bias, caused by prejudiced data in the family studies. Yet this orthodoxy was contradicted by what clinical researchers regularly witnessed in the families that they were working with.

A breakthrough occurred in the early 1990s, when researchers observed the unusual expansion pattern and ‘stretchy’ premutation on the gene connected to fragile X syndrome. This premutation presents as a ‘repeat’ in the pattern of the DNA: when passed on from parent to child, the premutation can either remain a premutation, or expand to a full mutation (expressed as a greater number of repeats). The full mutation (which expresses in the carrier as fragile X syndrome) is rare, but the premutation (where the carrier often appears clinically unaffected) is not. The sighting of the premutation is animatedly narrated by the scientists as ‘the most exciting thing in my scientific life’, a ‘catalytic’ event equivalent to the discovery of DNA itself. Observing small segments of DNA from the repeat region on a southern blot (a molecular biology technique used to isolate specific DNA sequences within a complex mixture), researchers were able to visually identify the growth in the premutation across several generations of the same family: ‘we could see the fragment growing from one generation to the next, we could actually see it changing in size on these X-ray films as we developed them. It was just an indescribable moment of eureka’. Despite the centrality of visual evidence to the plot, this pivotal discovery is told rather than shown (there are no graphics to assist the viewer in picturing a premutation, for example); the intention is to convey the affective thrill of discovery and the collaborative interpersonal relationships that facilitate the production of new knowledge, rather than necessarily to communicate scientific information to a lay audience.

*Tomorrow Belongs to Me* foregrounds collective endeavour, countering the once-dominant historiographical tendency to frame scientific progress as the achievement of isolated geniuses, offering instead a sustained narrative of collaboration, interdependence, and shared knowledge-making. The film demonstrates how scientific knowledge is actively made (rather than absolute), and how that process is shaped by human connection, instinct, and chance (qualities also associated with artistic practice). It opens by centring intuition and risk as a disembodied voice invites the viewer, ‘Let’s take a gamble. Let’s gamble that we understand the mechanism of anticipation.’ In an early scene, Christian Höweler (a Dutch neurologist whose work in the 1980s was crucial to understanding the genetics of DM1) talks of his ‘intuitive feeling’ that the orthodox position on anticipation was wrong, gesturing with his hands towards his body in a somatic expression of ‘gut feeling’. Later on, another scientist recalls ‘squirming in my seat’ with physical excitement at a conference where the findings on the fragile X mutation were presented. Here and elsewhere in the film, researchers’ body language and speech patterns eloquently signal the importance of instinct and emotion in their work, revealing how ‘scientific practice is entangled with affect’ (Casini 2023, p.96).[32] Silvia Casini has argued that ‘artists can contribute to digging up the history of […] embodied and emotional laboratory work’ and that foregrounding affectivity ‘might lead to a scientific practice where matters of concern and care are of equal footing to matters of fact’ (Casini 2023, p. 97).[32] By making manifest the embodied and affective qualities of lab work, *Tomorrow Belongs to Me* creates an opportunity for reflecting on the connections between scientific discovery and the communities that the research involves.

Whilst the film centres the clinical voice, family experience is an ever-present undercurrent that actively shapes the unfolding narrative of scientific discovery as the researchers’ testimonies reveal how they gradually realised that they needed to listen to families more closely in their work. In a key scene geneticist Stephanie L. Sherman – cited in the opening line of this article – recounts how assumptions about the transmission pattern of fragile X had shaped the questions that researchers were asking of the affected families. (The mutating gene that causes fragile X is linked to the X chromosome; a pattern of ‘normal transmitting males’ - later understood as men carrying a premutation - meant that researchers initially assumed that it was passed down the maternal line.) Having initially disregarded family histories involving paternal relatives with developmental delays, researchers eventually realised that they were witnessing a similar pattern over and again:

we began to slowly listen to the families, and hang our heads low and go back to those families and say ‘ok, now please tell me about your father’s side’.[1]

The centrality of lived experience to the project of (scientific) research is further underscored by a set of sculptures made by Donachie for the exhibition of *Tomorrow Belongs to Me* at the Hunterian Museum and Art Gallery in Glasgow in 2006 [Figure 3]. Titled with Sherman’s words, *Tell me about your father’s side*, these sculptures are lacquered wood and steel benches, designed to be sat on by museum-goers. Referencing the moment that scientists started to listen to the affected families, the sculptures also suggest the ‘right to rest’ and a politics of access for disabled people. A politics of access has more recently been articulated in the work of artists like Finnegan Shannon who makes exhibition seating a central part of their practice as a way of critiquing the ableist structures of the museum (Cachia 2025, pp.186-191).[50] Recognising that ‘futurity is customarily claimed for able-bodied people’ (p.51), Charlotte Jacob-Maguire[51] reads Shannon’s work as ‘a moment when what is possible is rendered visible’ (p. 51) and as an argument for a ‘different future, cripped and critical’ (p.52). Transposing this analysis to Donachie’s sculptural benches, we can read *Tell me about your father’s side* as an optimistic vision of a future in which people living with disabling genetic conditions might be more actively involved in the research that directly affects them.

The title *Tomorrow Belongs to Me* gestures towards that future. Referencing the song from the Broadway show and film *Cabaret* (1972), performed by a member of the Hitler Youth in a particularly disturbing scene that alludes to the racial policies and atrocities of Nazi Germany, the title invokes the historical associations between early genetic science and the eugenics movement. While acknowledging these histories, Donachie has also suggested a more positive reading of the project’s title in its intimation of a better future for people affected by myotonic dystrophy. This has come about at least in part as a result of improved communication between scientific research and patient groups. As one clinician reflected:

The section “tell me about your father” is particularly poignant and important, I think. This reminds us that good science involves seeking truth by making observations as they are, even if they don’t appear to make sense, rather than trying to contort our observations into pre-conceived models. It makes me think of a quote we were told at medical school, from Sir William Osler; “Listen to your patient, he is telling you the diagnosis.” (Dr. Mark Hamilton, from interview with the artist by email, 20 March 2016; cited Donachie 2016, p.111).[39]

The film concludes with Huntington’s Disease expert Nancy Wexler’s sobering comments on therapy and treatment; while myotonic dystrophy, fragile X and Huntingdon’s are diagnosable, they remain untreatable. The science is intellectually exciting, but ultimately of limited importance unless it translates to a better future for people living with these conditions:

Because I really think that [treatment] is really the point of doing all of this. You know it’s fascinating, it’s beautiful, it’s aesthetic, it’s gorgeous, but in the end people are dying.[1]

When *Tomorrow Belongs to Me* was premiered at the International Myotonic Dystrophy Consortium Meeting in Quebec City, Canada on 22 October 2005 (IDMC-5),^v^ the audience (predominantly composed of scientists) questioned this choice of ending, asking why conclude with something so negative when so much progress has been made? (Donachie 2016, p.40).[39] This response reveals delegates’ expectation that art should serve and celebrate medical science by communicating its successes to wider audiences. Rejecting this illustrative role, Donachie’s work insists on its independent critical positioning as art. Quietly drawing attention to the preconceptions and partialities that structure the production of biomedical knowledge, *Tomorrow Belongs to Me* can be productively understood as institutional critique; that is, as art practices which ‘do not situate themselves in opposition to the medical institutions but work within them, exploring – and challenging – their logic from inside the system’ (Johnstone 2023, p. 427).[52]

Sociologist Michael Anteby uses the figure of the interloper to reflect the challenges of field research, including the complexities of entering worlds that are typically inaccessible to outsiders.[16] Donachie’s interloping in the world of genetics research was made possible not by her personal experience as a member of a family affected by myotonic dystrophy, but by her professional status as an artist (Donachie 2016, p158).[39] Having been afforded privileged entry to this ‘closed’ world, Donachie used her skills and training as an artist to negotiate a shared space between (in her own words) ‘the “rigorous”, “expert” skills of an objective professional and the more subjective, unquantifiable experience/expertise of those directly affected by disability and loss (including myself)’ (Donachie 2016, p.161).[39]

Before the film was produced, Donachie presented her work at an international scientific meeting with participants from across the world, including a group of researchers who had worked on myotonic dystrophy for over twenty years, but who had never met a person with the condition. She refers to the work of the anthropologist James Clifford, who encouraged anthropologists to reflect on their relationship to the people they are studying:

What if someone studied the culture of computer hackers […] and in the process never “interfaced” in the flesh with a single hacker? Would the months, even years, spent on the Net be fieldwork? (Clifford 1997, p.61[53] in Donachie 2017, p.8)[54]

Donachie transposes this framework to consider her own contexts of medical research and artistic practice. How should researchers (whether scientists or artists) position themselves in relation to the subjects of their enquiry? What are the specific affordances or limitations of an explicitly first-person approach? And how might a collaborative approach enable different kinds of expert groups to come together to produce insights that might be richer or more nuanced than singular perspectives? (Donachie 2017, p.8).[54]

*Tomorrow Belongs to Me* epitomises the project of ‘critical interloping’ described in the introduction to this article. Mobilizing artistic research methods including ‘collaborative process and the coalition of expert knowledge’ (Donachie 2017, p.8)[54], *Tomorrow Belongs to Me* engages with, and brings into dialogue, the ‘expert cultures’ of biomedical science and lived experience. When I interviewed Donachie, she suggested that one of the most impactful aspects of her work on myotonic dystrophy has been to normalise contact between scientists and individuals living with the condition—something that was relatively unusual when she first began her research in the early 2000s. As part of a wider coalition of activity led by patient groups as well as creative practitioners and selected members of the scientific research community, Donachie’s research on myotonic dystrophy has played a significant part in shifting clinical perspectives. To give one concrete example of how this translates into practice, an increased emphasis on patient voices is now reshaping research priorities for future therapies, directing attention toward the symptoms that people living with the condition find most burdensome (rather than those that scientists find most interesting).

As *Tomorrow Belongs to Me* recognises, lived experience is a site of knowledge that usually sits ‘at the margins of genetic science’ (Donachie 2016, p.17)).[39] Artist-led research can be a valuable tool for articulating patients’ perspectives and bringing the two worlds of ‘scientific’ and ‘lived’ experience into dialogue with each other. If *Tomorrow Belongs to Me* approaches myotonic dystrophy predominantly through the lens of scientific knowledge, the film *Hazel*, explored in the next section of this article, focuses on everyday experiences of living with a multi-generational progressive condition, asking the viewer to consider relationships, structures of care, and the impact of the illness on normal domestic life.

### Hazel

‘Patients are experts too, and art can be a powerful method of articulating lived experience.’ (Donachie 2017, p.8)[39]

**Figure 4:**
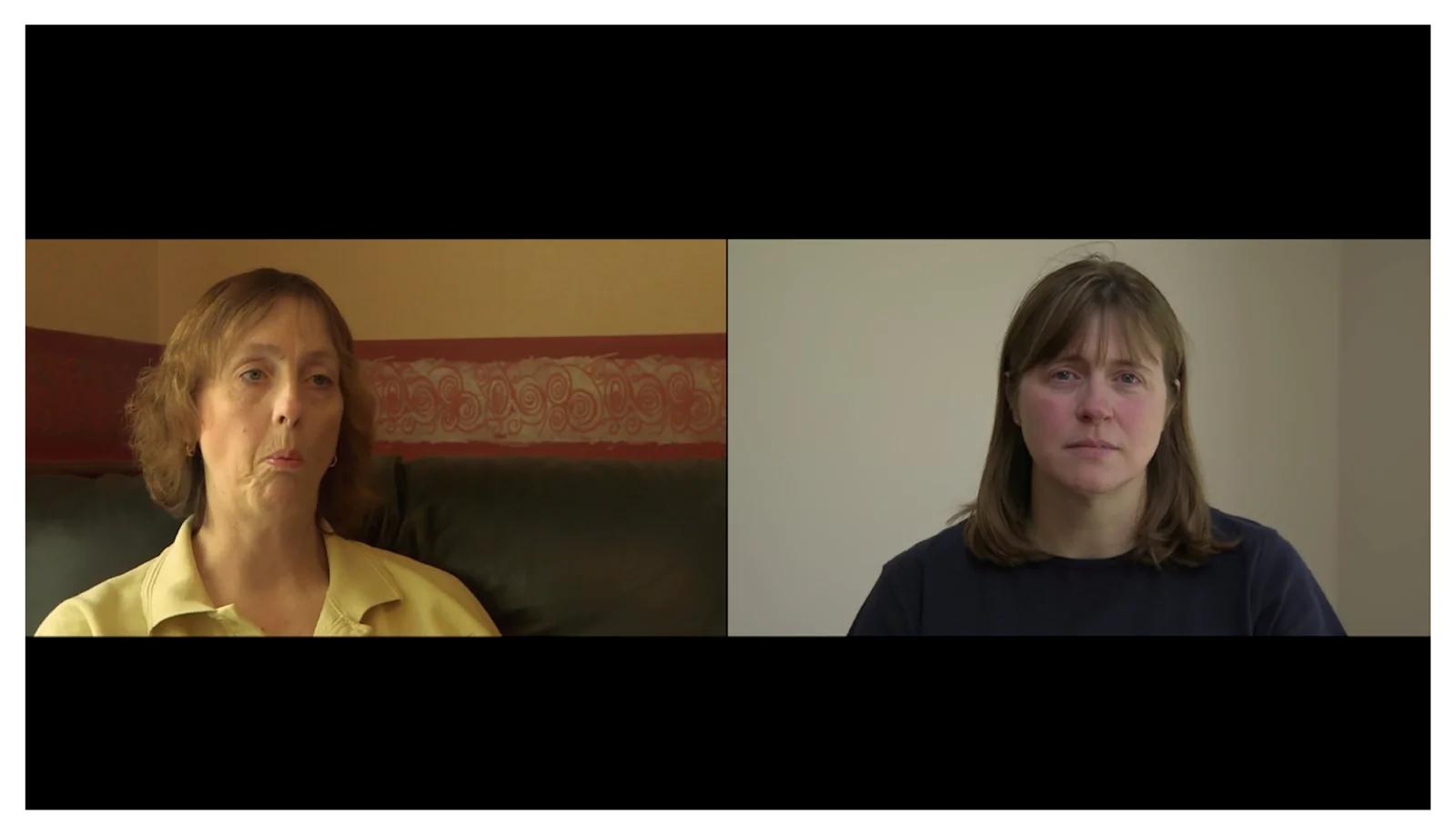
Detail from Jacqueline Donachie, *Hazel* (single screen, 2015). Image © the artist. *Hazel* can be viewed on the following link: https://discovery.dundee.ac.uk/en/publications/hazel-audio-video-installation/ [accessed 24 April 2026].

**Figure 5:**
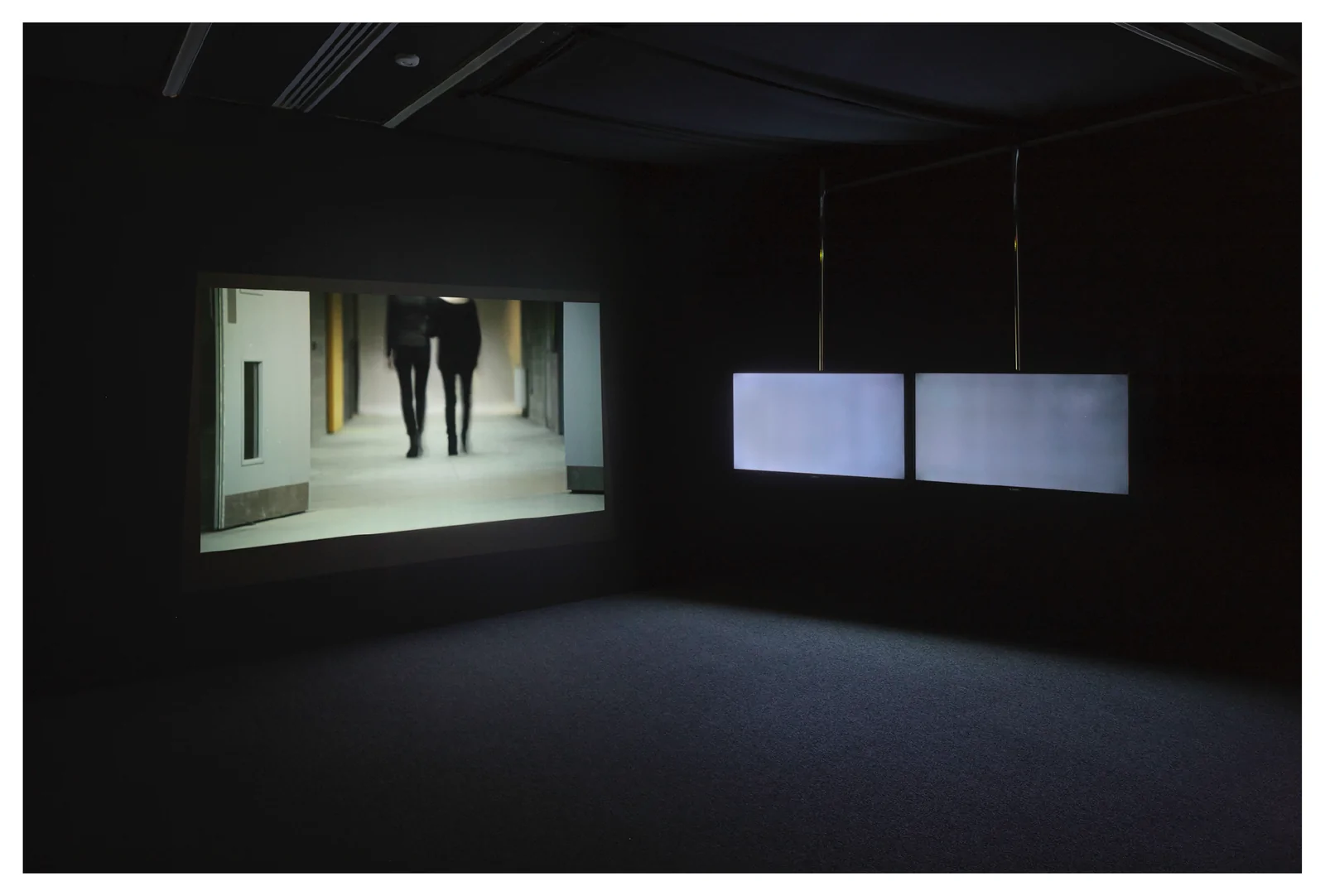
Jacqueline Donachie, *Hazel* (3-screen installation, 2015). Exhibition installation view, *Deep in the Heart of Your Brain*, Gallery of Modern Art, Glasgow, 2016. Photography Ruth Clark. Image © the artist.

*Hazel* is a short film (9 minutes 48 seconds) based on an extended set of interviews conducted by Donachie with sisters contacted via the UK Myotonic Dystrophy Patient Registry. Directly influenced by Donachie’s close relationship with her own sister, it focuses on pairs (and in one case a trio) of sisters where one is living with myotonic dystrophy and the other has been unaffected. Taking up the themes of the first line of Donachie’s artist’s book *DM* (‘All my life I’ve been told how much I look like my sister’), *Hazel* [Figures 4 and 5] explores ideas about family resemblance, and where that resemblance might end when one member is affected by an inherited illness that alters appearance. As Donachie put it: ‘when do you stop looking like your family and start looking like the symptoms described in a textbook?’ (Donachie 2017, p.7).[54]

A series of women appear on screen in pairs. Each sister is filmed in a separate frame: the sisters with myotonic dystrophy appear on the left-hand side of the screen, and the unaffected sisters on the right. Only the affected sisters speak. They candidly discuss the impact of myotonic dystrophy on their appearance: how faces drop, eyelids become hooded, and downwards-sloping mouths affect their ability to smile. One wishes that she was ‘a bit cleverer’ (an allusion to low educational attainment and low paid work that often affects the lives of people living with myotonic dystrophy); she also suggests that she would like her speech to be less slurred (as it affects people’s willingness to listen to her). The worst thing about myotonic dystrophy for her, however, is IBS (Irritable Bowel Syndrome) which is painful, inconvenient, and directly impacts her ability to participate in and enjoy everyday activities. They discuss difficult decisions around starting families of their own, and talk about their relationship with their parents (who may be ageing and experiencing mild symptoms of myotonic dystrophy themselves, often whilst caring for more severely affected members of the younger generations of the family). As each affected woman speaks, her unaffected sister gazes silently into the camera, their statue-like poses only occasionally disrupted by blinking or the barely perceptible rise and fall of their chest with their breath. This is a marked contrast to the more animated self-presentation of the speaking sisters; on several viewings I wondered if my gaze was lingering longer on the non-speaking (unaffected) sisters that the speaking (affected) ones, simply because I was drawn to the uncanniness of their stillness and silence.

Donachie recorded interview material with both affected and unaffected sisters; it was only during the editing process that she decided that the unaffected sisters would not speak in the film. Like *Tomorrow Belongs to Me, Hazel* is a tightly-edited distillation of a sizeable body of face-to-face ethnographic research. The interviews were conducted by Donachie with the women in their own homes, filmed by a cameraman in a deliberately low-tech set-up intended to be as un-intrusive as possible. Donachie collected over 8 hours of material, totalling approximately 70,000 words of transcribed text. As with *Tomorrow Belongs to Me*, the interview transcripts are available to read as part of an accompanying publication. Parts of the printed transcripts are heart-breaking: several of the women speaking describe experiencing stillbirths or neonatal losses of premature congenitally affected babies. Donachie made a deliberate decision not to include these more emotive aspects of the interviews in the final film, electing instead to communicate the ordinariness of living with the condition on a day-to-day basis. Grief and suffering are not overtly exhibited or aestheticized, but instead discretely etched on the barely-moving faces of the unaffected sisters. Although shown in parallel, the silent footage of the unaffected sisters was taken immediately after the interviews; Donachie asked each sister to remain in the room alone and look into the camera for five minutes as a way of discretely capturing the residual emotion from the preceding conversation (Donachie 2016, p.101).[39] What we see in the unaffected sisters’ faces is a quiet portrait of loss; a sibling relationship irrevocably shaped by the changes wrought by a progressive genetic condition.

Donachie chose to work specifically with sisters (rather than any other kind of sibling or family relationship) in response to her own experience. She and her sister appear in the final frames of the film: arm-in-arm and both dressed in black, they walk away from the camera, one taller, once slightly shorter, listing a little as she walks. Donachie describes the work as ‘a portrait of the ineffable loss of my sister’ (Donachie 2016, p. 81).[39] It is also a collective portrait, a testimony to a group whose stories are seldom shared. Dominant narratives about myotonic dystrophy typically centre on severely affected children (particularly when mobilised for fundraising purposes); middle-aged women are rarely represented. Yet many of these women carry a double burden of care, supporting children and even grandchildren with the condition, whilst also managing its progressive and often debilitating effects on their own lives.

Curious about how living with a progressive condition might shape women’s experiences of ageing and appearance, Donachie asked each sister to describe both her own looks and her sister’s, reflecting on the changes that have taken place since childhood. These reciprocal acts of description become a means of interrogating both family resemblance (a key theme in genetics research) and the ways in which disability shapes gendered experiences of aging. On several viewings, I found my eyes moving between the two halves of the screen trying to read the resemblance. When Donachie invited clinical experts to view the film (at the Gallery of Modern Art in Glasgow, where it was exhibited as part of a solo show in 2016), she asked them if they could see the family resemblance in the two sides: ‘They could only see the myotonic dystrophy running down the left-hand side’ (Donachie in Jeffrey 2017, p. 49).[40] Reading resemblance is contextual, shaped by our own histories, professional training and visual preconceptions.

*Hazel* offers a portrait of the ‘unseen family’, contextualising the patient beyond the clinical environment. Clinicians rarely encounter unaffected adult siblings; they might see parents or partners, but not siblings. To see *Hazel* as a clinician, therefore, is to get a sense of the ‘whole patient’, as attested to here:

I think one of the main things I took from *Hazel* was an insight into how dystrophy can impact the identity of an individual. As clinicians, we’re trained to look for the typical signs - such as facial weakness, droopy eyelids, even aspects of personality - that we associate with the disease. It is all too easy then to fall into the trap of defining the person by their illness; “another myotonic”. The effect of the silent sister - a range of different women from the general population - reminds us that the affected sisters are equally diverse, and encourages us to look beyond the superficial similarities of the disease and to see the individual. (Dr. Mark Hamilton, Clinical Geneticist, West of Scotland Genetics Services; interviewed by Donachie 20 March 2016 and cited in Donachie 2016, p.106).[39]

*Hazel* premiered at the International Myotonic Dystrophy Consortium Meeting (IDMC-10) in Paris on 12 June 2015, ten years after *Tomorrow Belongs to Me* had been presented to the same group in Quebec. The audience appeared moved but also unsettled; clinicians were particularly disappointed not to hear the voices of the unaffected sisters. Subsequent screening events for clinical staff in Newcastle and Glasgow reaped similar reactions. The clinicians’ frustration at the film’s silencing of the unaffected sisters – a group which they rarely encounter in their own work, and whose perspectives they were therefore eager to learn from – spoke to their own expectations of the artwork; that is, that it should ideally offer unambiguous testimony to lived experience and fulfil some sort of instructive function.

Donachie’s refusal to provide testimony or instruction is not a failure of the work, but a deliberate artistic decision. The considered withholding of information, for example, is an essential aspect of the aesthetics of the work. Donachie’s process often involves generating an extensive body of research (for example, hours of interviews) before paring her material back to produce a final artwork that is sparse and minimalistic, a poetic distillation of experience and meaning. The result is an artwork that is not didactic, but which – through its refusal to put everything on display for easy consumption – demands effort and engagement on the part of the viewer. If the viewer is willing to meet the work halfway, a much richer encounter can be had.

*Hazel* is an articulation of patient experience, and of the expert knowledge that this experience affords. It effectively and imaginatively communicates the experiences of a demographic of middle-aged women who have traditionally been absent from dominant representations of myotonic dystrophy. The idea that *Hazel* might offer a direct account of experience is understandably appealing to clinical audiences. Yet (as Stuart Hall pointed out) [55] no artwork can offer a truly authentic representation of people or events: culture is not a straightforward reflection of lived reality, but the framework through which individuals interpret, organise, and give meaning to their experiences.

## Conclusion

This article introduced the critical interloper, a figure well-practiced in accessing and engaging with different expert cultures, gathering together diverse perspectives and experiences to produce an original outcome that is more than merely the sum of its parts. Using Donachie’s work on myotonic dystrophy as a case study, it has proposed that the methods in which many artists are trained – dialogue, negotiation, holding multiple contradictory viewpoints together in a single space – are particularly well-suited to this task. In the contexts of healthcare and biomedical research, artists’ research methods are valuable tools for articulating the expertise of patients as well as scientists and clinicians, flattening epistemological hierarchies and bringing together different (often contradictory) forms of expert knowledge in productive conversation. Emphatically, in making this case I am not suggesting that artists’ practices should be assessed primarily or exclusively through the value that their work bring to clinical contexts. Such an approach not infrequently results in art which may hold some interest for an audience of healthcare professionals, but which is of limited significance and value in an artistic setting. One of the most compelling aspects of Donachie’s work is its legibility across multiple frameworks while preserving its status and specificity *as art*. This is not always easy or straightforward, something that I have tried to unpick at particular points in this article.

This article adopts a close reading of an extended body of work by a single artist, rather than a broader but less detailed survey of multiple practices. This decision reflects a commitment central to medical humanities scholarship: to attend closely to the nuance, specificity, and situated nature of interdisciplinary work at the intersections of clinical knowledge and lived experience. Such close attention is particularly important in relation to Donachie’s practice, where artistic methods engage with patients, clinicians, and medical infrastructures in ways that resist easy generalisation. Within the constraints of a single journal article, this depth of engagement would not have been possible had multiple artists been addressed. Recognising the limitations of an argument grounded in a single case study, future research might extend this approach to a wider range of artist-interlopers similarly working through collaborative, embodied, and relational forms of knowledge production between medicine and patients. Relevant examples include Catherine Baker’s work with young women with adolescent idiopathic scoliosis in collaboration with spinal surgeon Thanos Tsirikos;[56,56] Sofie Layton’s long-term collaboration with bioengineer Giovanni Biglino involving young patients with congenital heart conditions, their families, and clinical teams;[57–60] and Liz Orton’s work on medical imaging developed in partnership with radiologist Steve Halligan.[61–63] To engage adequately with the methodological, ethical, and affective complexities of these and related practices would require a book-length study. If, as Engebretsen et al. contend, the aim of a genuinely transformative or ‘translational’ medical humanities is to create ‘an interdisciplinary space where both medicine and the humanities challenge and inform each other’,[16] then artists must be understood not as supplementary figures within this space, but as uniquely positioned to lead its development and realisation.

## Footnotes

i In this article I use the term ‘artist-led research’ in preference to the more widely used ‘practice-led research’: the intention is to foreground artists as research leaders, and to highlight selected aspects of artists’ research methods as particularly pertinent to collaborative interdisciplinary enquiry.

ii The phrase ‘expert cultures’ is borrowed from the Cultural Negotiations of Science (CNoS) Research Group at Northumbria University. For more details visit the CNoS website: https://cnos.ac.uk

iii A brief note on the term ‘visual medical humanities’, which I have previously used as pragmatic shorthand for articulating the contributions that art practice, art history and visual culture studies might make to medical and health humanities research. I see two potential issues with this term. Firstly, it does not adequately capture the complexity of artistic practices such as Donachie’s that extend well beyond image-making and visual representation. Secondly, drawing distinctions between ‘visual’ and other forms of medical humanities scholarship risks further marginalising the kinds of practices that I would like to see more fully integrated into ‘mainstream’ medical humanities work.

iv The consortium ran from 1996 to 2002 and included the Arts Council of England (renamed Arts Council England in 2003), the Scottish Arts Council (a consortium member for the first year only), the British Council, the Calouste Gulbenkian Foundation, the National Endowment for Science, Technology and the Arts (NESTA), and the Trust.

v Following its debut at IDMC-5, *Tomorrow Belongs to Me* was exhibited as a solo show in the Hunterian Museum and Art Gallery in Glasgow in 2016, and has since been shown widely at scientific, medical and art conferences, testifying to its ability to reach a variety of disciplinary audiences.
